# 

**DOI:** 10.1192/bjb.2024.19

**Published:** 2024-10

**Authors:** Mihaela Bucur

**Affiliations:** Consultant psychiatrist at All Points North London, London, UK. Email: drbucur@apn.com

**Figure:**
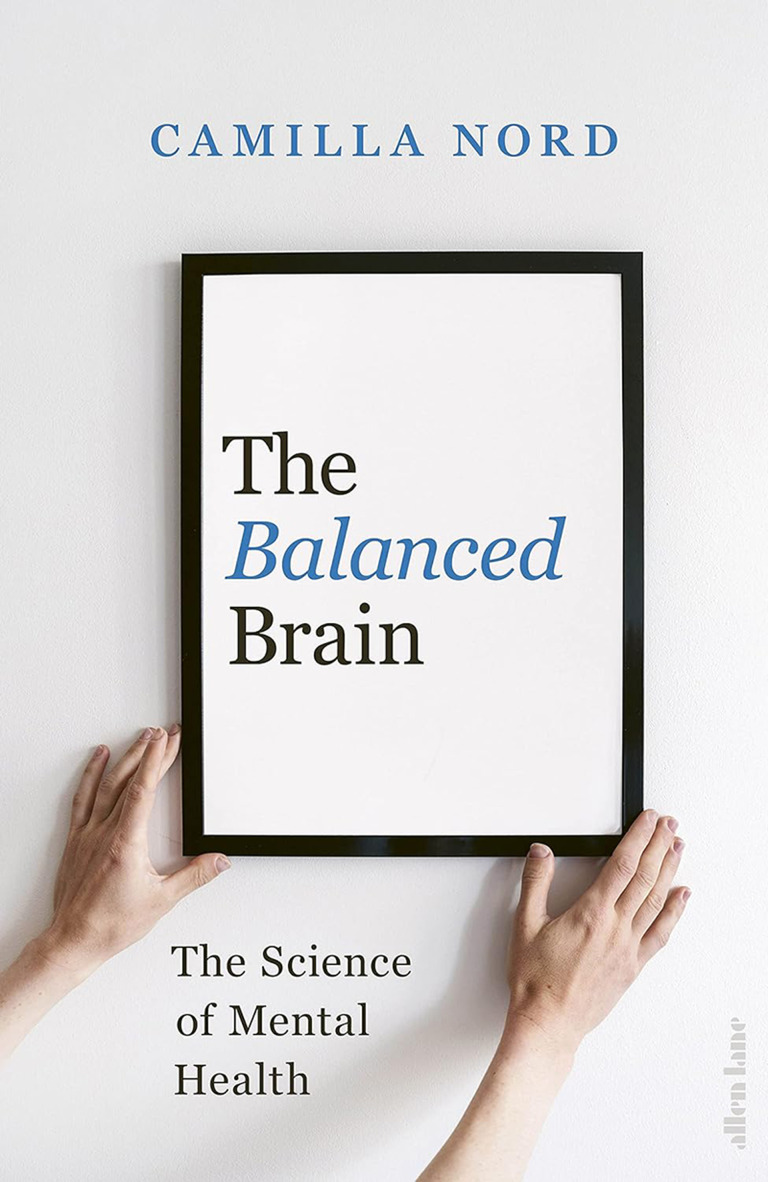

*The Balanced Brain: The Science of Mental Health* by Camilla Nord is an insightful book that offers a fresh perspective on achieving well-being. Nord, a renowned neuroscientist, provides a comprehensive exploration of the intricate brain processes that support mental health, shedding light on the latest advances in understanding and treating mental health conditions.

One of the most compelling aspects of Nord's book is her emphasis on the individualised nature of mental health. She advocates for treatments that target specific biological and cognitive patterns, urging a shift towards personalised approaches rather than relying solely on diagnostic categories. This perspective highlights the importance of personalised and tailored interventions for mental health conditions.

Nord delves into various processes involved in mental health, such as pleasure, pain, motivation and learning. By exploring the interplay between emotions and physical state, she reveals the complex relationship between mind and body and advocates for providing a more holistic approach to treatment. In addition, her examination of the impact of treatments on mental health outcomes, including the placebo and nocebo effects, provides valuable insight into the power of the mind in influencing mental well-being. She also explores the effects of antidepressants and psychotherapy, highlighting the importance of individual brain circuits in determining treatment effectiveness.

One of the standout chapters in the book is ‘Is there a mentally healthy lifestyle?’ Nord explores the growing evidence on the positive effects of exercise, nutrition and sleep on mental health, highlighting the role of resilience and self-efficacy. She also acknowledges potential pitfalls and side-effects of lifestyle changes, providing a balanced view of their benefits and challenges.

Throughout the book, Nord presents a wealth of scientific research and evidence to support her arguments. However, she offers this information in a clear and accessible manner, making it understandable to both professionals in the field and individuals seeking to improve their mental health. Nord's writing style is engaging, making the book a pleasure to read. *The Balanced Brain* is an empowering and enlightening book that challenges traditional notions of mental health and offers a fresh perspective on achieving well-being.

